# Sulfur Deprivation Modulates Salicylic Acid Responses via Nonexpressor of Pathogenesis-Related Gene 1 in *Arabidopsis thaliana*

**DOI:** 10.3390/plants10061065

**Published:** 2021-05-26

**Authors:** Steven Criollo-Arteaga, Sofia Moya-Jimenez, Martin Jimenez-Meza, Victor Gonzalez-Vera, Jessica Gordon-Nunez, Sol Llerena-Llerena, Dario X. Ramirez-Villacis, Pieter van ‘t Hof, Antonio Leon-Reyes

**Affiliations:** 1 Laboratorio de Biotecnología Agrícola y de Alimentos, Ingeniería en Agronomía, Colegio de Ciencias e Ingenierías, Universidad San Francisco de Quito USFQ, Diego de Robles y Vía Interoceánica, Quito 17-1200-841, Ecuador; edwsteve_7@hotmail.com (S.C.-A.); moyajimenezsofia@gmail.com (S.M.-J.); martinjimeza@gmail.com (M.J.-M.); victor.gonza85@gmail.com (V.G.-V.); gnunez.jessica@gmail.com (J.G.-N.); sllerena@usfq.edu.ec (S.L.-L.); dxramirez@usfq.edu.ec (D.X.R.-V.); 2 Colegio de Ciencias Biológicas y Ambientales, Instituto de Microbiología, Universidad San Francisco de Quito USFQ, Diego de Robles y Vía Interoceánica, Quito 17-1200-841, Ecuador; pvanthof@usfq.edu.ec; 3 Department of Biology, University of North Carolina at Chapel Hill, Chapel Hill, NC 27599-3280, USA; 4 Colegio de Ciencias Biológicas y Ambientales, Instituto BIOSFERA, Universidad San Francisco de Quito USFQ, Diego de Robles y Vía Interoceánica, Quito 17-1200-841, Ecuador; 5 Colegio de Ciencias Biológicas y Ambientales, Universidad San Francisco de Quito USFQ, Diego de Robles y Vía Interoceánica, Quito 17-1200-841, Ecuador

**Keywords:** sulfur, salicylic acid, plant defenses, NPR1, nutrition

## Abstract

Mineral nutrients are essential for plant growth and reproduction, yet only a few studies connect the nutritional status to plant innate immunity. The backbone of plant defense response is mainly controlled by two major hormones: salicylic acid (SA) and jasmonic acid (JA). This study investigated changes in the macronutrient concentration (deficiency/excess of nitrogen, phosphorus, potassium, magnesium, and sulfur) on the expression of *PR1*, a well-characterized marker in the SA-pathway, and *PDF1.2* and *LOX2* for the JA-pathway, analyzing plants carrying the promoter of each gene fused to GUS as a reporter. After histochemical GUS assays, we determined that *PR1* gene was strongly activated in response to sulfur (S) deficiency. Using RT-PCR, we observed that the induction of *PR1* depended on the function of Non-expressor of Pathogenesis-Related gene 1 (NPR1) and SA accumulation, as *PR1* was not expressed in *npr1-1* mutant and NahG plants under S-deprived conditions. Plants treated with different S-concentrations showed that total S-deprivation was required to induce SA-mediated defense responses. Additionally, bioassays revealed that S-deprived plants, induced resistance to the hemibiotrophic pathogen *Pseudomonas syringae* pv. DC3000 and increase susceptibility to the necrotrophic *Botrytis cinerea*. In conclusion, we observed a relationship between S and SA/JA-dependent defense mechanisms in Arabidopsis.

## 1. Introduction

For centuries, soil fertility practices have revolved around general indicators characterising water availability, effective disease control, and soil mineral content to improve crop productivity. Since then, many technological advances have developed a wide array of management practices, which without a doubt has opened agricultural barriers and provided access to unchartered territories, certainly pressured by the global growth of the human population. The nutritional balance, which indicates whether a soil maintains a proper balance based on the ratios between specific nutrients, is a significant factor of importance to manage soil fertility, as this balance is associated with the production and productivity of many crops [1].

Soil nutrient misbalance can be added to the long list of stressful situations with which plants must cope within their daily lives [2]. In addition, abiotic stress factors, including excessive drought, salinity, heat, or cold, are known to predispose plant disease susceptibility [3,4,5]. Sessile of origin, evolution forced plants to develop an extensive network of defense mechanisms to protect themselves [6]. Their innate immune system recognizes invading microorganisms through pattern recognition receptors (PPRs), which trigger many downstream defenses cascades, which in turn proved to be most effective for a plant’s survival [7,8].

Phytohormones salicylic acid (SA) and jasmonic acid (JA), and ethylene (ET) play an essential role in regulating and shaping the plant’s innate immune response [9]. The SA biosynthetic pathway, though not fully deciphered, is predominantly induced to fend off (hemi) biotrophic pathogens [10,11]. Traceable mobile molecules such as methyl-SA trigger a class of crucial early response defense genes [12]. The core of this frontline of early-induced defense genes is formed by genes encoding PATHOGENESIS-RELATED (PR) proteins, each with its distinct biochemical function and action spectrum [13,14]. Due to local and systemic accumulation, PR proteins are suggested to play critical parts in orchestrating a detrimental process called Systemic Acquired Resistance (SAR) [15]. Plants, pre-treated with inducing agents that mimic biotic stress stimuli, are capable to respond faster in their formation of adequate chemical or physical barriers against these attackers compared to untreated plants. Although various SA-binding proteins have been previously characterized, NPR1 plays a central role in SA signaling, as it binds SA and mounting a SAR response [16,17]. In addition to SA, other related compounds such as methyl salicylate (MeSA) or gentisic acid induce *PR1* expression [12,18].

In contrast, the JA response pathway is activated by herbivorous insects that chew on the leaves or infections by necrotrophic pathogens [19,20]. Plants have evolved a way to memorize these attacks and effectively use this pre-conditioned state and use it to their benefit in a process called Induced Systemic Resistance (ISR). Interestingly, JA and SA biosynthetic pathways have been reported to work antagonistically, as their regulation revealed a mechanism called “cross-talk.” Although SA and JA are the primary factors influencing plants’ defense mechanisms, other players were characterized to possess important immunity roles as ethylene, cytokinin, and auxins [21,22,23].

Over the years, several papers highlighted the critical link between nutrition and disease resistance [24,25,26], especially concerning N [27], P [28,29], K [29], Fe [30], and S [31]. For example, triggered by a pathogen attack, plants developed an iron retention strategy with increased iron uptake and redistribution within the cells to ultimately enhance the production of reactive oxygen species (ROS) [32,33]. Nitrogen has been proposed to be placed at the core of plant defense against pathogens since N forms influence physical barriers and disease resistance [34]. Moreover, N fertilization or N deprivation was shown to have a wide range of effects on plant defense physiology, influencing in some degree SA, JA, and ET biosynthetic pathways [27].

In addition to Fe and N, sulfur-containing stress compounds (SDCs), such as elemental sulfur, amino acids, and secondary metabolites rich in S, have been linked to plant immunity responses [35,36]. It has been demonstrated that JA-mediated biosynthesized SDCs served hands-on defense strategies against many invaders [37,38]. Sulfur is fourth in the list of essential macro elements mineral-based after nitrogen (N), potassium (K), and phosphorus (P), and it is vital for plant growth and development of all vascular plants, without exceptions [39]. Although plants can incorporate atmospheric H_2_S or SO_2_ [40,41], most of the sulfur is absorbed by plant roots in the form of sulfate (SO_4_). In *Arabidopsis thaliana*, several microarray data identified over 2000 sulfate-responsive genes involved in critical biological functions, including plant immunity [42,43]. Sulfur is reported to be relatively immobile in plants [44]. Hence, it is required to construct disulfide bridges with cysteine residues as precursors of relevant S-containing metabolites [31,44]. Its deficiency inhibits chlorophyll biosynthesis, affecting critical processes like electron transport, the redox cycle, and the production of essential secondary metabolites. Another mechanism in which plants evolved to cope with ambient sulfur deficiencies is the accumulation of other trace elements or even heavy metals such as cadmium [45,46].

Although many studies have published the importance of sulfur as a macronutrient for plant growth and resilience, only a few studies connect S to the concept of plant immunity. Sulfur has been claimed to increase the availability of other essential elements. Still, some authors go a step further, emphasising the role of Sulfur Induced Resistance (SIR), in which the application of S protects against plant diseases or improves general stress tolerance [31,47]. As early as 1897, Wheeler and Adams showed evidence that introducing elemental Sulfur (S^0^) to soil could help controlling common scabs in potatoes caused by *Streptomyces scabies* [48]. The response of sulfur-deficient plants has been connected to higher disease susceptibility, for example, *Pyrenopeziza brassicae* in oilseed rape or *Verticillium dahlia* in tomato. However, sulfur applications have been shown to reduce pathogens’ infections in several host plants when attacked by *Botrytis cinerea*, *Fusarium oxysporum*, and *Rhizoctonia solani* [31]. This induced resistance is linked to the production of reactive sulfur species (RSS), phytochelatins, and glutathione (GSH), a thiol compound, and a significant reservoir of reduced sulfur in plants [47]. Even though the exact biochemical mechanisms of how sulfur-derived compounds confer protection against fungal pathogens remain unclear to date, they are thought to negatively interfere with fungal redox reactions [31,49,50]. Moreover, S-Adenosyl Methionine (SAM) and the sulfur assimilation pathway directly regulates the ethylene biosynthetic pathway. Sulfur deficiencies, via SAM, could disturb plant immunity responses via ethylene. This indication points out that sulfur is essential for plant disease resistance [31,51].

Here, we investigated the role of mineral nutrients (N, P, K, Mg, and S) in the activation of plant defense responses by looking at the activation of the promotor of reporter genes fused GUS lines. Further, we deeply investigated S deficiency’s effect and its implications for innate plant immunity in *Arabidopsis thaliana*. Here, the expression of the SA marker gene *PR1* (well-characterized marker in the SA pathway) was monitored in plants that lacked sulfur or had excessive S concentrations. Then we examined the expression of the *PR1* gene in response to different S concentrations. Finally, Col-0 and *pad3* mutant plants were subjected to S-manipulated diets and challenged with *Pseudomonas syringe* DC3000 and *Botrytis cinerea* to determine disease resistance under S-deprivation and excess diets.

## 2. Results

### 2.1. The Absence of Sulfur Induces SA-mediated PR1 Expression

**Figure 1:**
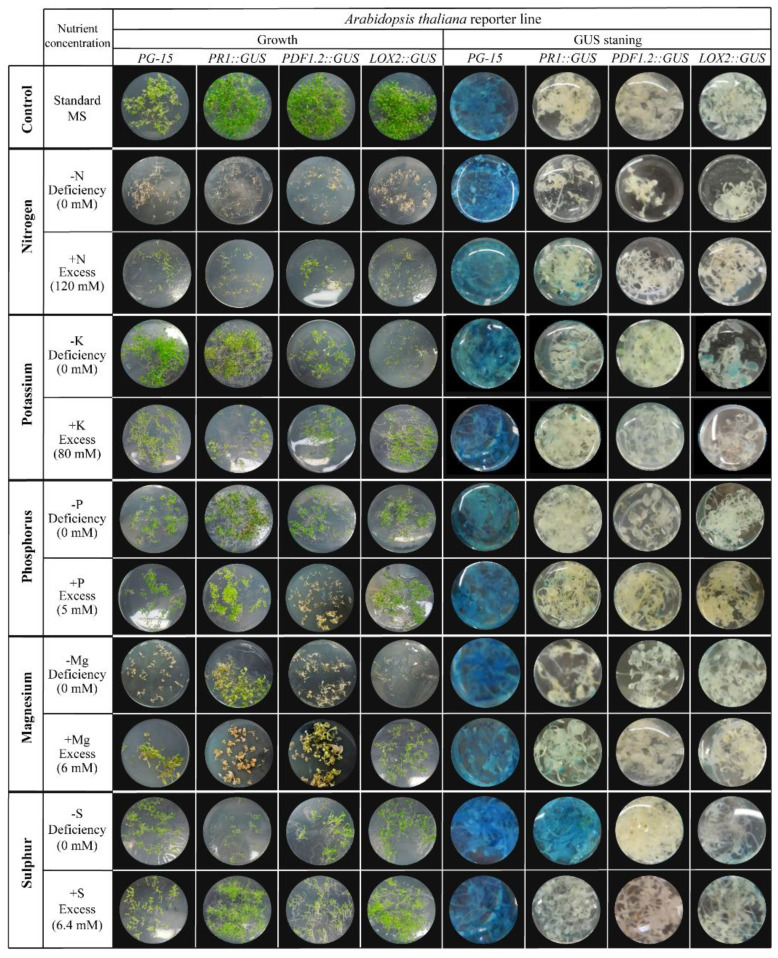
*Arabidopsis thaliana* responses to nutritional stress generated by the absence or excessive concentrations of nitrogen, potassium, sulfur, magnesium, or phosphorus under in vitro conditions. Growth and GUS-staining of 3-week-old plants of PG-15::GUS, PR1::GUS, PDF1.2::GUS, and LOX2::GUS reporter lines are shown. PG-15::GUS was used as a positive control for GUS-staining.

To assess the effect of macro-nutrient concentrations on the growth and stress responses in Arabidopsis, four different lines carrying the promoter of the marker gene fused to GUS (PR1::GUS for SA-mediated responses; PDF1.2::GUS for JA- and ET-mediated responses; LOX2::GUS for JA-mediated responses; and PG15:: GUS as a constitutive expressed control) were grown in vitro and subjected to MS treatments (control), either without (0%) or with excessive amounts (200% more for N and 400% for the rest of nutrients) of the macronutrient of interest (N, K, S, Mg, and P). The left panel of Figure 1 shows the development of seedlings after three weeks of incubation in standard MS medium (control; upper row in the figure), nutrient-lacking diet in MS medium (top row per treatment), or medium with excessive nutrient concentrations (lower row per treatment). All nutrient-manipulated treatments resulted in smaller seedlings in comparison to plants grown under complete-balance nutrient conditions. Plants treated explicitly with media, which either lacked potassium, sulfur, or phosphorous or contained excessive amounts of these nutrients, showed moderate growth. However, plants subjected to media manipulated with nitrogen or magnesium concentrations heavily affected seedlings’ growth. Both N-deprived and excessive-N media severely limited seedlings’ growth with a white-yellow coloration, compared to the larger, green seedlings cultivated in standard MS media. Moreover, a similar white-yellow coloration could be observed in tiny Arabidopsis seedlings in Mg-manipulated treatments.

Seedlings exposed to modified sulfur conditions showed leaves with either brown or chlorotic spots and yellow leaf borders, with thin, shorter stems (Figure S1).

Subsequently, all Arabidopsis seedlings were subjected to histochemical staining for GUS assay (Figure 1). The expression of the respective reporter gene was visualized by the blue staining of the leaf tissues. Constitutively expressed PG-15:: GUS control plants showed deep blue staining for seedlings grown in standard MS control media and all nutrient-manipulated treatments, indicating the transgene’s active expression even in the absence of these essential elements. In addition, some tiny and scattered blue leaf patches or no GUS staining were observed for all investigated macro-nutrients, except for sulfur. A different pattern could be seen when PR1::GUS reporter lines were stained for GUS activity after being cultivated in the total absence of sulfur, indicating high activation of *PR1* promoter in response to the lack of sulfur. 

No GUS staining of PR1 or PDF1.2 reporter plants was observed for standard or excessive sulfur regimes, emphasizing the unique response observed under sulfur deprivation. PDF1.2::GUS plants grown under the same feeding conditions showed no signs of blue staining in their tissues, indicating no upregulation of *PDF1.2*. Moreover, No blue staining was observed in the LOX2::GUS control reporter line. These patterns could be reproduced in three replicated assays. These results indicate that sulfur deprivation solely induced the salicylic acid-dependent *PR1* gene expression. On the other hand, *PDF1.2* and *LOX2*, which are marker genes for general plant defense responses mediated by jasmonic acid or ethylene, were not induced by sulfur deprivation.

### 2.2. Excess of Sulfur Inhibits SA-mediated PR1 Expression

**Figure 2:**
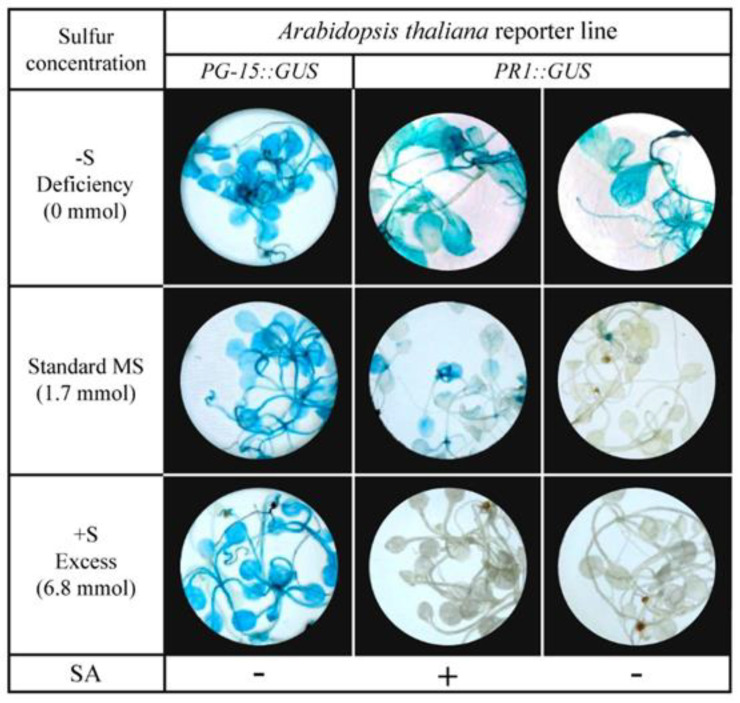
GUS histochemical staining assay of expression in PR1: GUS reporter line induced by deficient (-S) or excessive (+S) sulfur concentrations and an exogenous application of 0.5 mM SA 3-week-old in vitro plants. PG-15:: GUS plants were treated with milliQ water as a positive control.

To analyze the effect of sulfur on SA-mediated stress responses, we examined the additional effect of exogenous SA application on three-week-old *A. thaliana* seedlings, grown on standard, sulfur-lacking, or excessive sulfur solid MS medium. Figure 2 shows the GUS histochemical staining assay of PR1::GUS seedlings compared to control PG-15::GUS plants. 

GUS staining of PR1::GUS and PG-15::GUS seedlings at three different sulfur regimes without exogenous SA application confirmed similar results obtained in Figure 1. Sulfur-deprived PR1::GUS plants were the only seedlings displaying blue-stained leaf tissues, except for all PG-15::GUS control plants, which showed an intense GUS staining as expected, regardless of the sulfur concentrations. When plants were treated with 0.5 mM exogenous SA, *PR1* expression was induced in seedlings grown under optimal nutrient conditions. Sulfur-deprived PR1::GUS seedlings exposed to exogenous SA responded with similar GUS staining intensities, as observed in the absence of SA. In contrast, once sulfur-excess diets were subjected to exogenous SA-treated plants, no blue staining could be seen during the GUS activity assay. No detection of GUS expression indicates that the *PR1* defense gene remained inactive under these conditions. In conclusion, sulfur deficiency-induced *PR1* expression and contrast, SA-mediated *PR1* expression seemed to be inhibited when seedlings were treated with excessive amounts of sulfur.

### 2.3. Transient Expression of PR1 under Sulfur Deficiency

**Figure 3:**
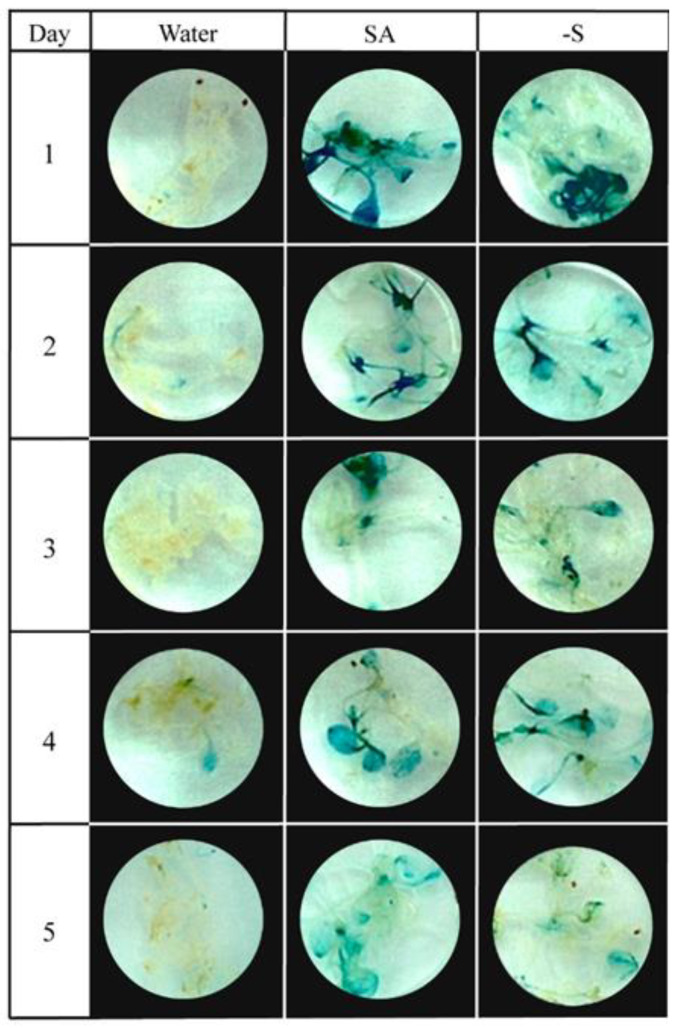
*PR1* expression for the first five days after treatment application on 3-week-old PR1::GUS in vitro plants. The activation of *PR1* with salicylic acid (SA) and sulfur deficiency (-S) was evaluated. Water treatment was used as a negative control.

To examine the robustness and longevity of the *PR1* defense gene induction, when trigger in the absence of sulfur, PR1::GUS plantlets were grown for three weeks in vitro on solid standard MS media before being exposed to three different treatments: only distilled water (Control), 3 min dipping in a solution of 0.5 mM SA solution +24 h standard MS (SA), and only 24 h sulfur-deprived MS (–S). After this period of treatment, all plants were transferred to distilled water. Every 24 h afterwards, for five consecutive days, five individual plants corresponding to every treatment were collected and transferred to the GUS staining solution. Figure 3 displays the five-day gradient of GUS staining patterns of this PR1::GUS plant.

The most robust PR1::GUS staining could be observed 24 h following the exogenous SA application. GUS activities in subsequent days demonstrated a gradual reduction in the expression of *PR1*, indicating that the plant gradually recovered from an initially experienced stress situation. A similar situation was observed on –S deprived plants.

### 2.4. A Total Absence of Sulfur Induces PR1 Expression

**Figure 4:**
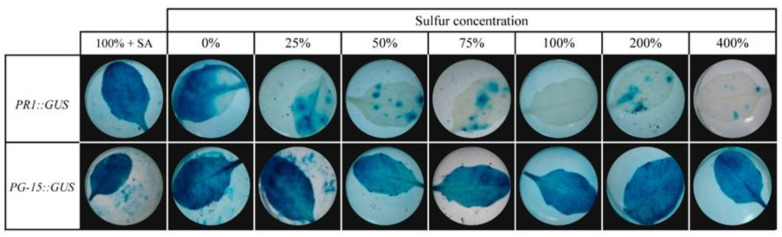
PR1 expression induced by application of different sulfur concentrations on five-week-old PR1::GUS plants. 100% corresponds to ½ MS standard (control) sulfur concentration. PG15::GUS line and SA application were used as positive controls.

To investigate the threshold when sulfur deficiency induces an SA-stress response, *A. thaliana* plants were subjected to more concentrations of modified sulfur diet treatments in a sulfur concentration gradient assay (Figure 4). Reporter lines PR1::GUS and constitutively expressed control PG15::GUS plants were grown on sterile river sand substrate under standard Hoagland solutions. After five weeks, these substrates were rinsed with distilled water for three days to remove the remaining Hoagland solutions. Next, to previously investigated standard (100%), 0% sulfur (-S), and 400% sulfur MS media, we also treated plants to liquid MS media with a partial sulfur deficient treatment containing 25% S, 50% S, and 75% S, and an additional 200% sulfur-surplus treatment. 24 h post-treatment, leaves of both reporter lines were subjected to a histochemical GUS activity staining to visualize ambient expression patterns. As shown in Figure 4, GUS-stained leaves indicated the highest induction of *PR1* activity in leaves of plants grown in the total absence of sulfur. Leaves of plants subjected to sulfur concentrations ranging from 25% up to 200% (compared to standard MS sulfur concentrations) demonstrated sparsely distributed, small blue-colored spots, in contrast to the total absence of staining standard sulfur nutritional conditions. This distribution pattern indicated local SA-mediated stress perceptions in small patches of neighboring cells in response to different levels of sulfur deficiency or excess, as it indicated the expression of *PR1*. Although newly added media with varying sulfur concentrations were added to the experimental design, plants did not show GUS staining patterns as previously obtained for sulfur-deprived plants (0%). This observation indicates that the total absence of S is needed for full-SA-mediated induction of plant defense responses.

### 2.5. SA Accumulation and Functional NPR1 Is Required for PR1 Expression Induced by Sulfur Deprivation

Earlier GUS-staining experiments on PR1 reporter lines showed a possible connection between sulfur deprivation and SA-mediated stress responses in *A. thaliana*. In search of additional evidence to confirm these observations, the expression of the *PR1* gene in response to sulfur-deprived diets or SAR-inducing control conditions was analyzed using RT-PCR. With this purpose, 3-week-old seedlings of the wild-type Col-0 and transgenic accession Nah-G and the mutant *npr1-1*, which modifications render them deficient in SA accumulation and signalling, respectively [52,53], were grown in vitro under optimal nutrient conditions. Plants were subsequently transferred to either a 0.5 mM salicylic acid solution for 3 min and 24 h incubation in standard MS solution (+SA), or 24 h incubation in liquid MS medium lacking sulfur (-S). 

**Figure 5:**
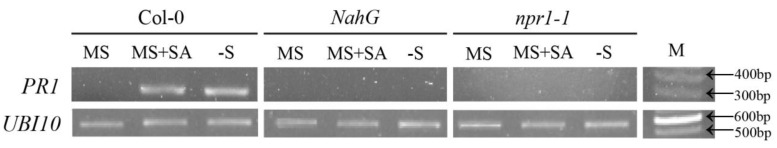
Gene expression analysis of *PR1* in wild-type (Col-0), NahG and *npr1-1* lines after treatment with ½ MS standard sulfur concentration and the sulfur deficiency (-S). UBI10 was used as a constitutive expression control gene. M, molecular weight marker.

The next day, leaves were harvested, and cDNA samples were synthesised from 1 µg of total RNA from each sample. As measured by RT-PCR, results of the *PR1* expression compared to the expression of the control gene UBIQUITIN 10 (UBI10) can be observed in Figure 5. Whereas *UBI10* showed bands of similar intensities for all analyzed samples, the induction of *PR1* expression in wild-type plants subjected to SA (MS + SA) was similar to plants under S deprivation (-S), as shown in previous experiments. In contrast, no *PR1* expression could be detected in control plants exposed to MS diet solution. None of the treatments induced the amplification of *PR1* in both NahG and *npr1-1* mutant plants, which confirmed that both SA and NPR1 are needed to induce SA-mediated responses under sulfur deprivation conditions.

### 2.6. Disease Resistance under Deficiency and Excess of Sulfur

**Figure 6:**
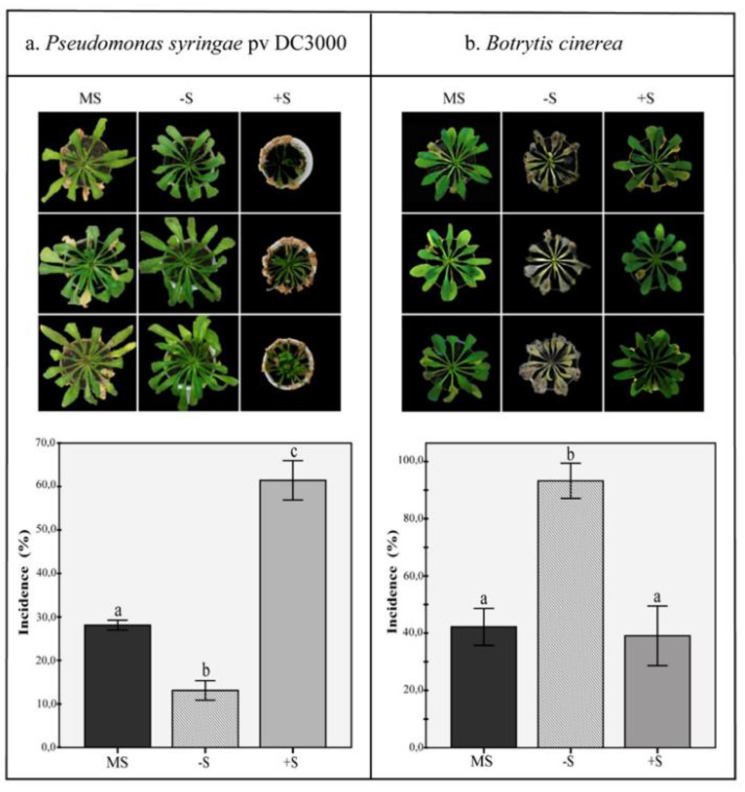
Disease development in (**a**) *Arabidopsis thaliana* wild-type (Col-0) after seven days of inoculation with *Pseudomonas syringae* pv. DC3000 and (**b**) *A. thaliana pad3* mutant after seven days of inoculation with *Botrytis cinerea*. Plants were treated with MS standard sulfur concentration (MS), sulfur deficiency (-S), and sulfur excess (+S). Disease symptoms are depicted in the upper panel, and the incidence is presented in the lower panel. The bars represent the mean of three independent replicates. Error bars indicate a standard deviation. Bars sharing the same letter are not significantly different (*p* < 0.05) determined by single-factor ANOVA with Tukey’s HSD test.

Earlier observations implied a direct link between the induction of SA-mediated stress responses to prevalent sulfur deficiencies. It raised questions on how these sulfur-deprived plants would react towards disease resistance. Thus, plantlets were grown in autoclaved river sand substrates under standard Hoagland nutrient regimes. Six weeks later, all river sand substrates were rinsed for four days with distilled water and subsequently watered with either standard (MS), sulfur-lacking (-S) or sulfur-surplus (+S) MS media and subsequently challenged with either the hemibiotrophic bacterium *Pseudomonas syringae* pv. DC3000, or the necrotrophic fungus *Botrytis cinerea*. Figure 6a shows the evaluation of disease symptoms of wild-type Col-0 plants, seven days after drop-inoculation with a *P. syringae* pv. DC3000 bacteria suspension and exposed to the various sulfur diets. Plants sulfur-deprived diets showed much fewer symptoms of infection by *P. syringae* pv. DC3000 than the mock control plants (Figure 6a).

In contrast, Col-0 plants exposed to excessive sulfur concentrations displayed strong disease symptoms, with necrotic and chlorotic leaves in all inoculated plants. These observations confirm the activation of the SA-mediated defense response in sulfur-deprived plants, resulting in plants resistant to Pst DC3000. Contrastingly, plants experiencing diets with excessive amounts of sulfur did not mount an adequate SA-mediated response, resulting in disease establishment by the hemibiotrophic pathogen within seven dpi.

To analyze S-deprived plants’ effect on resistance to *B. cinerea*, *pad-3* mutant plants were grown under similar conditions as earlier during *Pst* DC-3000 bioassay. Mutant *pad-3* has constitutively low camalexin levels (phytoalexin), leading to high susceptibility to *B. cinerea*. Mutant *pad3* has been used for being an excellent model for bioassays with necrotrophic pathogens since its resistance is not compromised on hormonal signaling pathways [54,55,56]. Figure 6B shows six-week-old *pad3* mutant plants, seven days after drop-inoculation with *B. cinerea* spore inoculum. Compared to the mock control plants inoculated with distilled water, *pad3* mutant plants were severely infected by *B. cinerea* under sulfur-deprived nutrient conditions. However, *pad3* mutant plants exposed to excessive amounts of sulfur were visually unchanged by this pathogen’s presence and showed healthy fresh green plants at the end of our experiment.

## 3. Discussion

Like all other organisms on earth, from the smallest bacteria to whales and majestic sequoia trees, all plants strongly depend on the availability of macro- and micronutrients for rapid growth and successful development of their roots, stems, leaves, and flowers [57]. The influence of nutrition on plants’ physiological responses has been studied intensively since the early 19th century. Though initially developed by Sprengel, Justus von Liebig [58] earned his fame for describing the “*Law of the Minimum*”, which explained the effects of individual nutrients on crop species. This law indicated that plants absorb essential nutrients from their immediate environment, only in proportion to other nutrients [58]. Therefore, nutrient deficiencies seem to directly orchestrate how well plants incorporate other nutrients, regardless of their abundance. To date, many studies have addressed the topic of plant nutrition and nutrient cycling processes. Results have shown that the total number of inorganic mineral elements, essential for an adequate plant development, ranges between 14 and 18 members [5,59,60]. Although each of these nutrients has been functionally characterized regarding their effects on plant physiology or organismal development, surprisingly, little is known about the role nutrients playing a role in plant innate immunity mechanisms [5,61]. 

In our experiments, transgenic Arabidopsis plants carrying the promoter of the marker gene *PR1, PDF1.2,* or *LOX2* fused to GUS, were subjected to diets containing a standard, deficiency, or excess concentration of nitrogen, phosphorus, potassium, magnesium, and sulfur. Using this experimental setup, we could identify the link between nutrient changes and SA- and JA-mediated responses (Figure 1). In addition to sulfur, which will be discussed further, changes in N and K concentration led to small changes in marker expression. In our study, excess of nitrogen in the medium led to low but detectable induction of *PR1* activation compared to its control having standard levels of N, similarly to studies reviewed by Mur et al. [34]. In tobacco, nitrate induces SA-mediated responses such as *PR1a* activation, accumulation of SA, stimulation of hypersensitive response, production of polyamides such as spermine/spermidine, and enhance resistance to the hemibiotrophic pathogen *Pseudomonas syringae* pv. tabaci [62]. In our study, K-deprivation conditions lead to the low but detectable activation of *LOX2* using the GUS reporter lines (Figure 1), meaning that the absence of K has a relation with JA-mediated responses, as previously review by Wang et al. [63]. Among many functions in plant growth, K-deficiency induced jasmonates-related compounds such as oxylipins and led to the accumulation of plant defenses such as glucosinolates [64]. Taking all together, it demonstrates that changes in nutrient composition could influence the response of defense hormones and that our experimental setup could pick up those chances similarly as presented in our studies. 

The current study investigated the response of model plant *Arabidopsis thaliana* towards deficiencies, sufficiency, and excessive amounts of sulfur. The main result clearly showed that sulfur-deprived diets strongly induced the SA-regulated stress marker gene *PR1*. In vivo by GUS-reporter line assays and in vitro by measuring *PR1* expression levels using RT-PCR demonstrated SA-mediated defense responses inducing *PR1* upon S-deprivation a similar extent of plants exposed to exogenous SA applications. Thus, these results revealed that the induction of SA-mediated defense responses directly affects the complete lack of sulfur in their diet. This phenomenon has not been described before in literature. Moreover, modified diets with partial S-deficiencies or excessive S amounts up to 400% of the standard MS dose did not result in systemic induction of *PR1* expression than the plants that lacked total access to a sulfur source (Figure 4). 

Some studies presented evidence for interactions between sulfur and SA-dependant defense signaling pathway. As reviewed by Bloem [31], the application of sulfur, mainly as a form of sulfate, could reduce disease infection, expressed as disease index (DI), to several necrotrophic, obligate biotrophic, and hemibiotrophic pathogens in several plant species such as Arabidopsis, *Brassica napus*, *Zea mays*, *Solanum lycopersicum*, *Vitis vinifera*, and *Triticum aestivum.* For example, S applications led to enhanced plant resistance against the necrotrophic pathogens: *Alternaria brassicicola* [65], *Sclerotinia sclerotiorum* [66], *Bipolis maydis* [66], *Rhizoctonia solani* [67], and *Rhizoctonia cerealis* [66]. These observations are in concordance to our study where the excess of S reduced *Botrytis cinerea* infection, and the absence of S increases its susceptibility in *Arabidopsis thaliana*.

In the literature, the general application of S seems to induced resistance to both SA and JA defense pathways to control necrotrophic and biotrophic pathogens, which contrasts our observation of enhanced susceptibility to *Pst* DC3000 in plants under S deficiency conditions. In agreement with this, Wang et al. [66] showed increasing amendments of sulfur to the plant species *Chenopodium amaranticolor* and *Gossypium* sp L. increased plant’s susceptibility to the biotrophic pathogen Tobacco Mosaic Virus (TMV) and the hemibiotrophic pathogen *Verticillium dahlia,* respectively. This will mean that the excess of sulfur would inhibit both SA-mediated responses and resistance, leading to increase susceptibility to (hemi) biotrophic pathogens, phenomena similarly observed in our study since plants were susceptible to *Pst* DC3000 when treated with an excess of S. 

Moreover, in an independent study, the authors described results from field studies where sulfur applications changed the metabolic defense response (cysteine and glutathione) of oilseed rape [68]. However, correlations with these observations with resistance and differences in disease progression against the hemibiotrophic pathogen *Pyrenoperiza brassicae* were not found [68]. Therefore, it seems that sulfur-induced responses are determined by environmental soil conditions or changes in the microbiome after applying sulfur. Recently, it was demonstrated that soil application of elemental sulfur (S^0^) changed the bacterial communities in the absence of phosphorus, leading to yield improvement in durum wheat by enhancing the microbially mediated nutrient mobilization [69]. These are open questions that have to be answered in the future [70].

Ball et al. [71] described a direct link between the expression of an arsenal of stress-responsive genes in response to changes in the biosynthetic metabolism of glutathione, a key exponent of a sulfur-derived metabolite. Rausch and Wachter [37], in turn, claimed that the glutathione metabolic pathway might be related to the NPR1 redox potential. Interestingly, NPR1 plays a crucial role as a major regulator of SA-mediated responses to pathogens, including the upregulation of the PATHOGENESIS-RELATED (PR) gene family [72]. Inactive NPR1 resides in the cytosol when plants are not attacked, but the presence of SA triggers the reduction of NPR1 into monomers [73]. These monomers are translocated to the nucleus, where induces the expression of SA-dependent genes [74].

However, the exact mechanisms linking the sulfur nutritional status and SA-mediated upregulation of *PR* genes in *A. thaliana* remain mostly unclear [49]. The longevity and robustness of the *PR1* expression, as analyzed in the absence of sulfur in treatments, indicated the highest expression levels around 24h post-treatment, followed by a gradual fading of this signal until it disappeared after the fourth day (Figure 3). Similar results were published by Ohshima et al. [75], where the PR1a:: GUS reporter line was sprayed with exogenous SA, and the PR1a induction signals were followed in subsequent days in GUS-staining assays. This peak of *PR1* expression around 24 h post-treatment could be explained by the mRNA accumulation process, which is locally seen in a minute to hours range. It is important to mention that when SA-signaling spreads to plant tissues further away from the infection site, it is essential to mention that the systemic component can take up to 24 h and is preceded by a burst in reactive oxygen species [76,77,78]. 

Nah-G and *npr1-1* mutant plants did not show any signs of *PR1* expression caused by any of the tested treatments, confirming SA and NPR1 protein’s role as a critical component of SAR signalling and its relationship with sulfur [52]. Earlier observations indicating a direct link between the induction of SA-regulated stress responses in Arabidopsis in answer to prevalent sulfur deficiencies raised questions on how *A. thaliana* would react towards pathogen infections in the absence of sulfur. To test the consequence of sulfur misbalance on defense responses of *A. thaliana* under pathogen attack, different lines were challenged with either *Pseudomonas syringae* pv. DC3000 or *Botrytis cinerea*. Results shown in Figure 6 confirmed that the lack of sulfur effectively influenced the outcome of Arabidopsis-pathogen interactions. When S-deprived Col-0 plants were challenged with hemibiotrophic bacteria *P. syringae* pv, DC3000, all plants remained healthy and showed no visible disease symptoms (Figure 6a). However, mock-treated plants on standard diets showed moderate disease symptoms, while Col-0 plants with access to excessive amounts of sulfur displayed heavily diseased plants. These results delivered evidence that *P. syringae* pv. DC3000 was unable to establish a successful infection in sulfur-deprived Col-0 plants. Moreover, this outcome suggested that sulfur-deprived diets can enhance the SA-mediated immune response of Arabidopsis towards hemibiotrophic pathogens.

PAD3 mutation leads to infection by necrotrophic pathogens when challenged, for example, with the necrotrophic fungus *B. cinerea* [56]. However, when S-deprived pad-3 plants, the infection intensifies, negatively affecting pad3 plants (Figure 6b). However, mock-treated plants on standard diets showed moderate disease symptoms, while pad3 mutant plants with access to excessive amounts of sulfur were visually no disease by the presence of the pathogen. Moreover, these findings confirmed observation in earlier studies, which suggested a relationship between the interference of defense genes by JA and, promoting resistance against necrotrophic pathogens [37]. Preliminary evidence indicating a hormonal cross-talk between SA- and JA-regulated defense responses shed light on why sulfur-deprived pad-3 plants could not mount an adequate defense strategy against *B. cinerea*. Cross-talk between SA and JA pathways has been studied during plant-microbe interactions and the molecular level, mainly when SA activates the suppression of JA responses [79,80]. It has been shown that this balance between SA and JA, can alter the outcome of plant response. For instance, JA responses triggered by necrotrophic pathogens such as *Alternaria brassicicola* and *Botrytis cinerea* or herbivory insects like *Pieris rapae* and *Frankliniella occidentalis* could be effectible suppressed by the external application of SA [81]. Spoel et al. [82] demonstrated that the induction of SA-mediated responses led to enhanced susceptibility to *A. brassicicola* infection. This study found a similar situation when sulfur deficiency triggered induced resistance to Pst DC3000 and enhanced susceptibility to *Botrytis cinerea*. 

**Figure 7:**
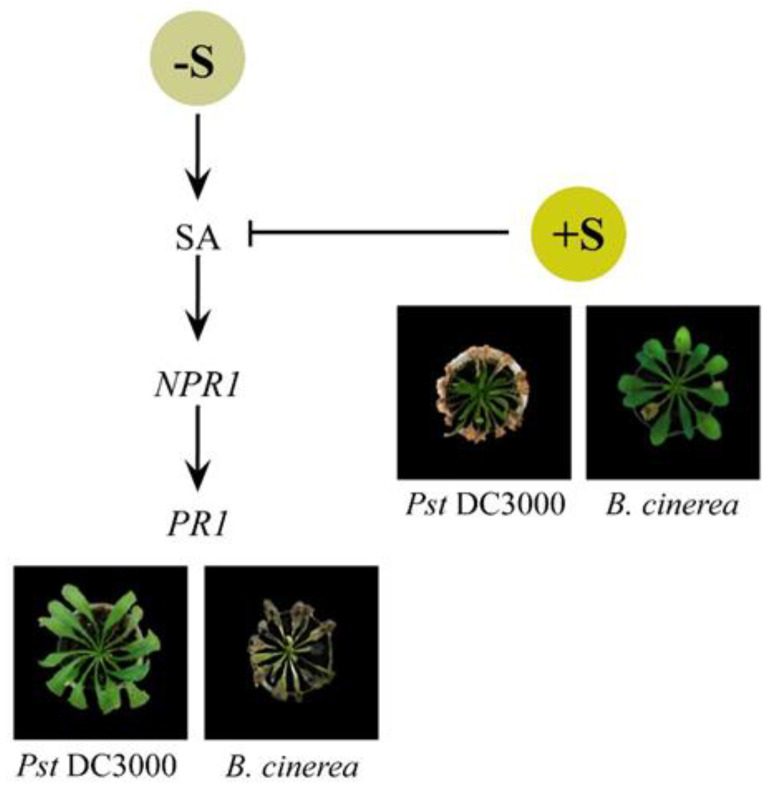
A schematic model is integrating the effect of sulfur on innate plant immunity mechanisms against *Pseudomonas syringae* pv. DC3000 (Pst DC3000) and *Botrytis cinerea* using *Arabidopsis thaliana* as a model.

Based on new insights generated by this study, we propose the following simplified model, integrating the sulfur-dietary status and plant defense mechanisms to predict the outcome of Arabidopsis-pathogen interactions (Figure 7). This model demonstrates that, on the one hand, the absence of sulfur promotes SA-mediated defense elements, including the upregulation of *PR1*, rendering plants susceptible to *B. cinerea* and resistant towards infection of *P. syringae* pv. DC3000. On the other hand, high S concentrations lead to less SA biosynthesis and more JA, promoting plants to fend off necrotrophic pathogens like *B. cinerea* successfully. However, this situation comes with a cost, as they become more susceptible to (hemi)biotrophic invaders. 

Although our results indicated a control mechanism of SA-mediated stress genes in response to sulfur deprivation in *A. thaliana*, here, we cannot exclude the possibility of a more complex signalling network. A balance between both SA and JA and the pathways controlled by these phytohormones could also be involved. Based on earlier transcriptome analyses of gene expression patterns in sulfur-deprived *A. thaliana* plants, Nikiforova et al. [83] reported complex systematic responses, including JA pathway elements. This correlation between JA-regulated signalling responses and ambient sulfur concentrations has been supported by published evidence on various sulfur-derived compounds [84]. Thus, our study contributes to the intrinsic knowledge of the sophisticated immune system and plant nutrition. In this perspective, new strategies have to be developed using plant nutrients to reduce plant diseases from an agricultural perspective. 

## 4. Conclusions

In conclusion, it has been demonstrated that S-deprivation induces SA-mediated *PR1* gene expression, and excess of S suppresses SA-responses. The total absence of S in the diet is required for SA induction. Functional NPR1 is needed for *PR1* induction since expression was not observed in *npr1-1* mutant. S-deficiency induced SA-mediated resistance to *Pst* DC3000 and induced JA-mediated susceptibility to Botrytis, indicating that the absence of sulfur could be implicated via SA-mediated suppression of JA responses. When S-surplus was applied to the plants, the opposite occurred, treatment with S-excess induced resistance to *B. cinerea* and further induced susceptibility to *Pst* DC3000. Overall, indicating that sulfur’s modification could manipulate the cross-talk between SA and JA resistance in plants.

## 5. Material and Methods

### 5.1. Plant Material

*Arabidopsis thaliana* ecotype Columbia-0 (Col-0), *npr1-1*, Nah-G, PR1::GUS (NASC: N6357), PDF1.2::GUS, LOX2::GUS (NASC: N57953), and the constitutive β-glucuronidase-expressing line PG15 (35S::GUS) were previously described and kindly provided from the laboratory of Corné Pieterse at Utrecht University [85].

#### 5.1.1. Growth Conditions and Nutrient Treatment for *A. thaliana* In Vitro Assays

All in vitro assays used seeds previously surface-sterilised using the vapour method described by Clough and Bent [86]. Sterilized seeds were subjected to three different treatments (standard diet, minus nutrient, excess of nutrient) for nitrogen (N), potassium (K), sulfur (S), magnesium (Mg), and phosphorous (P), to assess the effect on the stress response. The concentration established by Murashige and Skoog [87] for in vitro media culture was considered the standard for each nutrient. Nutrient deficiency media (-N, -K, -S, -Mg, and -P) contained all the elements and standard concentrations of regular MS, except for the deficient nutrient (0 mM). Nutrient excess media (+N, +K, +S, +Mg, and +P) contained all the elements and standard concentrations of regular MS, except for the excessive nutrient which had either 2 times (+N) or 4 times (other nutrients) the MS standard concentration. Table 1 shows nutrient concentrations and treatments. All media were supplemented with 2% (*w*/*v*) sucrose, solidified by 0.8% (*w*/*v*) Bacto-agar (BD, Difco, USA). Moreover, pH was corrected to 5.6 with KOH 0.1M before autoclaving (15 min, 121 °C). After applying the sterilized seeds on the different media, the plates were transferred to a growth incubator room (25 ± 2 °C, 25–30% humidity) under 24 h light photoperiod for three weeks. Light intensity used was between 120 and 180 µmol/m^2^/s.

#### 5.1.2. *A. thaliana* In Vitro Assays: Sulfur Stress and SA-Mediated Pathways Evaluation

We monitored the *PR1* expression using GUS reporter lines (PR1::GUS and PG15) under sulfur deficient diets to evaluate the relation between sulfur-related stress and SA-mediated pathways. Histochemical staining assays evaluated the GUS activity. Simultaneously, RT-PCR measured the activation of SA-mediated response by a sulfur deficiency in Col-0, *npr1-1*, and Nah-G plants. Sterilized seeds of PR1::GUS, PG15, Col-0, *npr1-1*, and Nah-G were grown on plates containing 1/2 MS medium and supplemented with 2% (*w*/*v*) sucrose, solidified by 0.8% (*w*/*v*) Bacto-agar (BD, Difco, USA), and pH was corrected to 5.6 with KOH 0.1 M before autoclaving (15 min, 121 °C). All plates were incubated under the same controlled conditions as described above. After three weeks, the seedlings were harvested and transferred to a six-well cell culture plate, with five plants per well with 1 mL of one of the following sulfur treatments: MS with standard sulfur concentration, sulfur deficiency (-S) or sulfur excess (+S) (Table 1). Plants were exposed to these treatments for 24 h, and then the solutions were removed. Finally, plants were rinsed with Milli-Q water. Subsequently, 1 mL of either 0.5 mM SA (Sigma-Aldrich) or mock solution was applied to each well for 3 min and washed again with Milli-Q water. In the final step, 1 mL of Milli-Q water was applied to each well, and the plants were harvested after 24 h. Only PR1::GUS and PG15 (GUS histochemical staining) and Col-0, *npr1-1*, and Nah-G (RNA isolation and RT-PCR) plants from an additional well were harvests every consecutive day for five days to measure the duration for the *PR1* expression under these sulfur treatments.

### 5.2. A. thaliana In Vivo Assays: Greenhouse Conditions and Nutrient Treatments

Before in vivo assays, black plastic pots (60 mL) were filled up to 1 cm from the top with autoclaved river sand (45 min at 121 °C). A single seed of *A. thaliana* PR1::GUS or PG15 reporter lines was planted just below the surface in the center of each pot and transferred to a cold room (4 °C) for 24 h. After this cold period, pots were transferred to the greenhouse, where they were maintained for five weeks under natural light conditions (12 h light/12 h dark), at 24 ± 4 °C, with a relative humidity of 60–70%. Plants were watered every other day with 10 mL of Milli-Q water, and additionally, once a week with 10 mL of standard Hoagland solution [88]. To assess the intrinsic effect of sulfur on the expression of *PR1* in vivo, five-week-old Arabidopsis plantlets, which were previously irrigated with standard Hoagland solution, were rinsed with miliQ water for four days until nutrients washed off. After that, plants were subjected to new irrigation regimes of MS solutions with modified sulfur concentrations ranging from 0 mM (0%) to 6.4 mM (400%) and maintained under the same greenhouse conditions for the next 24 h. After 24 h, the fourth leaf was removed, rinsed with milli-Q water, and subjected to GUS histochemical staining.

### 5.3. Histochemical GUS Assays

After the nutritional treatments, GUS reporter lines seedlings (4-5 per well) or leaves (1 per well) were placed in a 24-well plate [85]. Next, 1 mL of the GUS staining solution (1 mM X-Gluc, 100 mM NaPi buffer, pH 7.0, 10 mM EDTA and 0.1% (*v*/*v*) Triton X-100) was added to each well [89]. For GUS activity determination, the plates were subjected to a partial vacuum (-15 Bar) for 1 h in a glass vacuum desiccator attached to a vacuum pump. Afterwards, they were covered by lids, sealed with parafilm, and transferred to an incubator at 37 °C for 2-3 days. After this incubation period, the GUS staining solution was removed, and the plantlets and leaves were de-stained using 70% ethanol for 24 h. Blue-stained tissues were examined and subsequently photographed with a digital camera (Canon EOS DSLR) [85].

### 5.4. RNA Extraction and RT-PCR

Total RNA from seedlings was isolated following the protocol of Van Wees [90]. According to the manufacturer’s protocol, cDNA was synthesised from 1 µg total RNA using SuperScript™ cDNA Synthesis Kit (Invitrogen™, Massachusetts, USA). The expression of SA marker gene *PR1* (AT2G14610) was examined by RT-PCR using gene-specific primers (PR1-F: 5′-TTCTTCCCTCGAAAGCTCAA-3′; PR1-R: 5′-ACTTTGGCACATCCGAGTCT-3′). *AtUBI10* was used as a constitutive control gene (UBI-F: 5′-AACAATTGGAGGATGGTCGT-3′; UBI-R: 5′-CAAGTTTCGCAGAACTGCAC-3′). 

The RT-PCR reaction contained 2 µL of cDNA, 1x PCR Buffer 4 mM MgCl_2_ 0.4 mM of each deoxyribonucleotide triphosphate 0.4 mM of each primer, and 5U of Platinum™ Taq DNA Polymerase (all reagents purchased at Invitrogen™, Massachusetts, USA), resulting in a final volume of 50 µL. Reactions were performed using the MultiGene™ OptiMax Thermal Cycler (Labnet, New Jersey, USA). The following thermal cycling program was selected for all RT-PCR reactions: 95 °C for 10 min; 26 cycles of 95 °C for 60 s, 50 °C for 30 s, and 72 °C for 60 s. RT-PCR products were visualised on a 2% agarose gel in 1x TAE buffer and stained with SYBR Safe (Invitrogen™, Massachusetts, USA). TrackIt™ 100 bp DNA Ladder (Thermo Fisher Scientific, Massachusetts, USA) was used as a reference, and the expected size for *PR1* and *UBI* was 346 bp and 573 bp, respectively.

### 5.5. Evaluation of Pseudomonas syringae and Botrytis cinerea Disease Resistance on Sulfur-Manipulated Diets

To assess the effect of sulfur on Arabidopsis disease immunity, *A. thaliana* Col-0 and *pad3* mutant seedlings were grown for five weeks in autoclaved river sand under standard Hoagland irrigation conditions as previously explained. Nutrient solutions containing deficient (-S), standard (MS), or excessive (+S) concentrations of sulfur (Table 1) were applied a day before pathogen inoculations.

Five-week-old Col-0 and *pad3* plantlets subjected to different sulfur treatments were exposed to two different pathogens, *Pseudomonas syringae* pv. *tomato* DC3000 (Molecular Plant Sciences Institute, Edinburgh, UK), and *Botrytis cinerea* strain (Agricultural and Food Laboratory, USFQ, Ecuador). This *B. cinerea* strain was previously isolated from the petals of infected roses [91]. *P. syringae* DC3000 bioassays were performed according to Van Wees [90], whereas *B. cinerea* bioassays followed the instructions published by Van Wees, Denby, and Corwin [92,93,94].

*P. syringae* DC3000 was cultured in 100 mL King’s B medium at 28 °C, shaken at 120 rpm for 24 h. The next day, the culture was centrifuged, and the pellet was re-suspended in 10 mM MgSO_4_ solution. Bacterial density was adjusted to OD600 of 0.1 (2.5–5 × 10^7^ UFC/mL) with a Jenway 731,501 7315 UV/Visible spectrophotometer (Jenway, USA). Bacteria were inoculated by dipping the whole plant for three seconds in the bacterial suspension. Inoculated plants were incubated at 22–25 °C under high humidity (between 90 and 100%), and the disease incidence was evaluated seven days post-infection (dpi). Leaves were photographed with a digital camera (Canon EOS DSLR).

Spores of *B. cinerea* were obtained from sporulating mycelia on two-week-old PDA plates, incubated at 25°C under continuous light. The fungal mycelium was gently brushed to release the conidia. The inoculum was collected in sterile Petri dishes containing liquid PDB and adjusted to 8 × 10^5^ conidia/mL (Neubauer counting chamber). Every second Arabidopsis leaf (counted from the rosette up) was drop-inoculated (5 µL) with the previously prepared spore solution, while control plants were inoculated with 5 µL of PDB media without spores. A total of five plants was used per treatment. Arabidopsis plants were incubated at 22–25 °C under high humidity (between 90% and 100%), and the disease incidence was monitored after seven days, according to Van Wees et al. [92]. Diseased plants were photographed with a digital camera (Canon EOS DSLR).

## Figures and Tables

**Table 1:** Nutrient concentration (N, K, S, Mg, and P) for each treatment (a deficiency, standard, and excess).

	Deficiency (mM)		Standard (mM)		Excess (mM)	
Nitrogen	-N	0	MS	60	+N	120
Potassium	-K	0		20	+K	80
Sulfur	-S	0		1.6	+S	6.4
Magnesium	-Mg	0		1.5	+Mg	6
Phosphorous	-P	0		1.25	+P	5

## Supplementary Materials

The following are available online at https://www.mdpi.com/article/10.3390/plants10061065/s1, Figure S1: Phenotypic characteristics of in vitro plants grown under sulfur deficiency (Right) versus its control (Left).
