# Supplementary material for: Sulfur Deprivation Modulates Salicylic Acid Responses via Nonexpressor of Pathogenesis-Related Gene 1 in Arabidopsis thaliana
Source: Plants (Basel). 2021 May 26;10(6):1065. doi: 10.3390/plants10061065 (PMC8230334; doi:10.3390/plants10061065)
Supplement: Supplementary file 1 [file plants-10-01065-s001.zip › plants-1209391-supplementary.pdf]

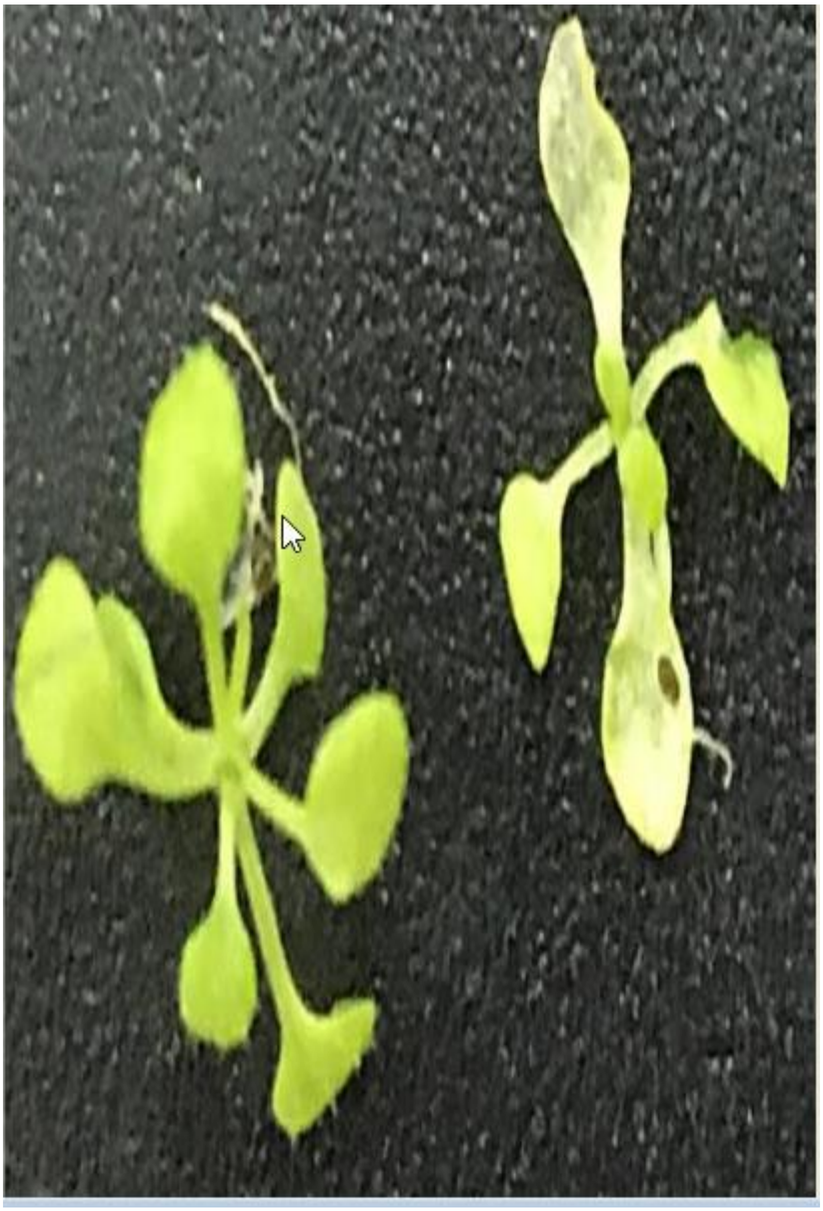

Figure S1: Phenotypic characteristics of *in vitro* plants grown under sulfur deficiency (Right) versus its control (Left).
